# Clinical Significance of Intestinal Fungal Overgrowth: Integrating the Gut Mycobiome into Modern Gastroenterology

**DOI:** 10.3390/microorganisms14061365

**Published:** 2026-06-19

**Authors:** Jisoon Im, Kyucheol Lee, Sang-Hoon Lee, Soohwan Jung, Kyu-Nam Kim, Jiyoung Lee

**Affiliations:** 1 Department of Family Practice and Community Health, Ajou University School of Medicine, 164 World Cup-ro, Yeongtong-gu, Suwon 16499, Republic of Korea; freejack777@naver.com; 2 Plastic Surgery, Cheongdamrui Plastic Surgery, Jeju 63083, Republic of Korea; 3 GreenCross I-MED Gangnam Center, Majestar City Tower 1, 4F, 12 Seocho-daero 38-gil, Seocho-gu, Seoul 06655, Republic of Korea; 4 Philip Medical Center, 10 Hwangsaeul-ro, 311 beon-gil, Bundang-gu, Seongnam-si 13590, Republic of Korea; 5 Biochemistry & Molecular Medicine, GW Cancer Center, School of Medicine & Health Sciences, The George Washington University, Ross Hall 437C, Washington, DC 20037, USA

**Keywords:** intestinal fungal overgrowth, gut mycobiome, candida, dysbiosis, intestinal permeability, microbiome interactions

## Abstract

Intestinal fungal overgrowth (IFO) is an increasingly recognized yet underexplored component of gut dysbiosis with potential implications for gastrointestinal and systemic disease. While bacterial microbiota have historically garnered research attention, recent advances in sequencing technologies have highlighted the importance of the gut mycobiome in maintaining intestinal homeostasis. Disruption of fungal–bacterial balance, particularly involving *Candida albicans*, *C. tropicalis*, and *C. glabrata*, may contribute to symptom generation through immune activation, epithelial barrier dysfunction, biofilm formation, and the production of toxic metabolites such as acetaldehyde and candidalysin. Emerging clinical evidence suggests that IFO is associated with persistent gastrointestinal symptoms, including bloating, abdominal discomfort, and altered bowel habits, particularly in patients who do not respond to conventional therapies targeting bacterial overgrowth. Furthermore, fungal dysbiosis involving *Malassezia restricta* and *Saccharomyces cerevisiae* has been associated with inflammatory bowel disease, metabolic disorders, and systemic immune dysregulation; however, the nature and directionality of these relationships remain incompletely understood. Despite increasing recognition, the diagnosis of IFO remains challenging due to a lack of standardized criteria and validated non-invasive tools. Therapeutic strategies, including antifungal agents such as fluconazole and nystatin, as well as microbiome-targeted interventions, show promise but require further validation. This review provides a comprehensive synthesis of current evidence regarding the epidemiology, pathophysiology, clinical manifestations, diagnostic challenges, and therapeutic implications of IFO, with particular emphasis on species-specific mechanisms. Recognition of the intestinal mycobiome as a potentially important component of gut health may provide new perspectives for understanding gastrointestinal disorders and inform future precision medicine approaches.

## 1. Introduction

The gastrointestinal tract is a complex ecosystem in which microorganisms coexist in a dynamic and tightly regulated equilibrium [1]. While bacterial communities have traditionally dominated microbiome research, fungi represent a critical yet comparatively understudied component of this ecosystem. Despite their relatively low abundance, fungal organisms exert significant influence on host physiology, immune regulation, and inter-microbial interactions [2].

Intestinal fungal overgrowth (IFO), including small intestinal fungal overgrowth (SIFO), is defined as a disruption of this ecological balance characterized by excessive proliferation of fungal organisms beyond commensal levels. Importantly, IFO reflects not only an increase in fungal burden but also a shift toward pathogenic behavior influenced by environmental and host-related factors. Among the most clinically relevant organisms are *Candida albicans*, *C. glabrata*, and *C. tropicalis*, each of which exhibits distinct pathogenic strategies including epithelial invasion, persistence, and biofilm formation [3,4,5]. Additional taxa such as *Saccharomyces cerevisiae* and *Malassezia restricta* have also been implicated in immune modulation and inflammatory processes [6].

Under physiological conditions, fungal populations are tightly controlled by host immunity and microbial competition. However, disruption of these regulatory mechanisms may favor fungal expansion and has been hypothesized to influence disease-related processes through alterations in microbial ecology and host responses.

This narrative review was based on a literature search of PubMed, Scopus, and Web of Science for studies published through May 2026. Search terms included “intestinal fungal overgrowth,” “small intestinal fungal overgrowth,” “gut mycobiome,” “fungal dysbiosis,” “Candida,” “biofilm,” “intestinal permeability,” “inflammatory bowel disease,” and “antifungal therapy,” together with related terms relevant to fungal–host interactions, diagnosis, and treatment. Particular emphasis was placed on primary human studies, mechanistic investigations, and recent high-quality reviews relevant to intestinal fungal dysbiosis. Given the absence of standardized diagnostic criteria and the heterogeneity of the available evidence, the present work was conducted as a narrative review rather than a systematic review.

## 2. Defining IFO: Current Concepts and Controversies

One of the major challenges in the field of IFO is the absence of universally accepted diagnostic criteria and standardized definitions. Although the term IFO is increasingly used in the literature, it encompasses a spectrum of fungal-related intestinal alterations that are often inconsistently defined. Fungal colonization refers to the presence of fungal organisms within the gastrointestinal tract without evidence of pathogenic activity or clinically relevant symptoms. In contrast, fungal dysbiosis describes qualitative or quantitative alterations in the intestinal mycobiome composition, diversity, or ecological balance, which may or may not be associated with disease. Fungal overgrowth generally implies excessive fungal proliferation beyond expected physiological levels, potentially accompanied by host immune activation, mucosal dysfunction, and symptom generation. Importantly, the term IFO is used in this review as a conceptual framework describing clinically relevant fungal expansion within the gastrointestinal tract rather than a formally established disease entity. Accordingly, the findings discussed throughout this review should be interpreted within the context of ongoing scientific uncertainty and evolving diagnostic paradigms.

## 3. Epidemiology and Risk Factors

The epidemiology of IFO remains incompletely defined due to the variability of diagnostic methodologies and the absence of standardized criteria. Nevertheless, limited clinical evidence suggests that fungal overgrowth may be detected in 20–30% of patients with unexplained gastrointestinal symptoms [7]. Specifically, a landmark retrospective study by Erdogan and Rao [7] evaluated a highly selected tertiary care cohort of 150 symptomatic patients presenting with chronic, refractory gas, bloating, and abdominal pain, reporting a 26% prevalence rate predominantly driven by *C. albicans*. In that study, SIFO was operationally defined using small intestinal aspirate culture—the current reference standard—with a quantitative threshold of ≥10^3^ CFU/mL. However, this figure is subject to substantial epidemiological variability and should not be generalized to the broader population. Operational differences in culture media and incubation parameters across laboratories can significantly affect fungal recovery rates. Furthermore, tertiary care populations introduce a profound selection bias due to a higher prevalence of gastrointestinal dysmotility and extensive prior exposure to proton pump inhibitors and broad-spectrum antibiotics, which inherently promote fungal proliferation compared to the general population.

An additional challenge in interpreting the epidemiology of IFO is the substantial variability of the intestinal mycobiome across populations. Emerging evidence indicates that fungal community composition is influenced by dietary habits, geographic location, environmental exposures, cultural practices, and host-related factors [8,9,10]. For example, dietary patterns rich in carbohydrates may favor the expansion of *Candida* species, whereas differences in food consumption, sanitation, climate, and microbial exposures may contribute to regional variation in fungal composition. Consequently, findings derived from specific populations may not be directly generalizable to other geographic or demographic settings. These factors should be considered when interpreting studies of fungal dysbiosis and may partially explain inconsistencies observed across the current literature.

The major risk factors associated with IFO, including medication exposure, metabolic conditions, and lifestyle influences, are summarized in Table 1. Antibiotic exposure represents the most significant risk factor, primarily through disruption of bacterial communities that provide colonization resistance [11,12]. This ecological imbalance creates a permissive environment for fungal expansion, particularly favoring species such as *C. albicans* and *C. tropicalis* [13]. Proton pump inhibitors further contribute by reducing gastric acidity, thereby facilitating fungal survival and colonization in the upper gastrointestinal tract [14].

Metabolic conditions such as diabetes mellitus promote fungal proliferation through hyperglycemia-induced alterations in host immunity and microbial ecology; *C. glabrata* demonstrates particular adaptive advantages in this environment [15]. In addition, host lipid metabolism has been implicated in shaping fungal composition, particularly through its influence on lipid-dependent organisms such as *Malassezia restricta* [16]. Immunosuppression is a major predisposing factor for fungal overgrowth. Impairment of cell-mediated immunity, particularly dysfunction of the Th17 axis, reduces mucosal antifungal defense and facilitates uncontrolled expansion of opportunistic fungi such as *Candida* species [17]. This is particularly evident in patients with underlying immunodeficiency or malignancy or in those receiving immunomodulatory therapies. Corticosteroid use further exacerbates susceptibility to fungal overgrowth through multiple mechanisms, including suppression of innate immune responses, inhibition of neutrophil function, and impairment of cytokine signaling pathways [18]. In addition, corticosteroids may alter mucosal barrier integrity, thereby creating a permissive environment for fungal colonization and persistence. Gastrointestinal motility disorders constitute another important risk factor. Impaired motility leads to luminal stasis, which promotes microbial accumulation and prolonged contact between fungal organisms and the intestinal epithelium [7]. This environment facilitates both fungal proliferation and biofilm formation, contributing to persistent dysbiosis. Reduced bile acid activity has also been implicated in fungal overgrowth. Bile acids possess intrinsic antimicrobial properties and play a critical role in regulating microbial composition within the gastrointestinal tract [19]. Alterations in bile acid metabolism, whether due to liver disease, dysbiosis, or dietary factors, may reduce antifungal activity and favor fungal persistence. Dietary patterns, particularly high intake of simple carbohydrates, provide readily fermentable substrates that support fungal growth and metabolic activity [20]. *Candida* species exhibit pronounced metabolic adaptability to carbohydrate-rich environments, which leads to increased production of fermentation byproducts that may contribute to symptom generation. Alcohol consumption represents an additional contributing factor through its effects on both fungal metabolism and epithelial integrity. Furthermore, ethanol metabolism by *Candida* species results in the production of acetaldehyde, a toxic metabolite that disrupts tight junctions and increases intestinal permeability [21]. In addition, alcohol-induced alterations in the gut microbiota may indirectly promote fungal overgrowth. Finally, chronic illness and hospitalization are associated with a heightened risk of fungal dysbiosis [22]. These conditions are frequently accompanied by factors such as polypharmacy, antibiotic exposure, nutritional imbalance, and immune dysregulation, all of which contribute to the disruption of microbial homeostasis and increased susceptibility to opportunistic fungal expansion.

## 4. Pathophysiological Mechanisms

**Figure 1:**
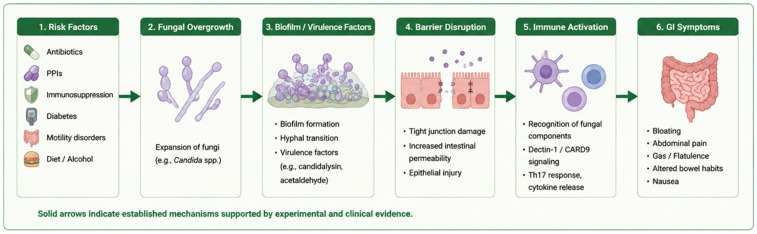
Established Mechanistic Pathway of Intestinal Fungal Overgrowth (IFO). Predisposing factors, including antibiotic exposure, proton pump inhibitor use, immunosuppression, diabetes mellitus, gastrointestinal dysmotility, and dietary influences, may promote fungal expansion within the gastrointestinal tract. Fungal overgrowth may subsequently contribute to biofilm formation, virulence factor production, epithelial barrier disruption, and activation of antifungal immune pathways, ultimately resulting in gastrointestinal symptom development. Solid arrows indicate mechanisms supported by experimental and clinical evidence.

The currently recognized pathophysiological framework of IFO is summarized in Figure 1. As illustrated, disruption of bacterial–fungal homeostasis may promote fungal expansion, virulence factor production, epithelial barrier dysfunction, and activation of antifungal immune pathways, ultimately contributing to gastrointestinal symptom development. These processes occur within the broader ecological context of the intestinal microbiome, which is characterized by highly dynamic and complex interkingdom interactions between fungal and bacterial communities. Such interactions influence fungal colonization, virulence, and host responses in a species-dependent manner. The species-specific contributions of key fungal organisms implicated in IFO are summarized in Table 2.

Under physiological conditions, bacterial populations exert a suppressive effect on fungal organisms through competition for nutrients, antimicrobial peptide production, and local immune response [23]. However, antibiotic-induced dysbiosis disrupts this ecological balance by depleting commensal bacterial populations, thereby reducing colonization resistance and permitting the expansion of opportunistic fungi [24]. Experimental and clinical studies have consistently demonstrated that such disruptions favor the proliferation of *C. albicans*, which can transition from a commensal yeast form to an invasive hyphal phenotype [25,26]. This morphological switch is a key virulence determinant, as hyphal forms exhibit enhanced adherence, tissue invasion, and resistance to host defenses [27].

The pathogenicity of *C. albicans* is further amplified by its ability to undergo phenotypic switching in response to environmental cues, including pH changes, nutrient availability, and host immune signals [28]. Hyphal transformation is associated with the expression of virulence genes encoding adhesins, proteases, and cytolytic factors, which enable the organism to penetrate epithelial barriers and establish infection [3]. This transition also facilitates the formation of structured microbial communities, which play a critical role in disease persistence [29].

In addition to *C. albicans*, *C. tropicalis* has attracted increasing attention as a potential contributor to intestinal pathology. Notably, *C. tropicalis* has been shown to form polymicrobial biofilms in conjunction with bacterial species such as *Escherichia coli* and *Serratia marcescens* [30]. These findings, derived from in vitro and in vivo models, indicate that fungal overgrowth is not an isolated phenomenon but rather occurs within a broader context of microbial network disruption [31,32]. The polymicrobial biofilms formed by *C. tropicalis* exhibit enhanced structural integrity, resistance to antimicrobial agents, and increased inflammatory potential compared to mono-species biofilms [33]. These observations suggest that synergistic fungal–bacterial interactions may contribute to chronic intestinal inflammation; however, the extent to which these interactions directly influence disease pathogenesis in humans remains uncertain.

Host immune responses to fungal overgrowth constitute a central pathophysiological component. Fungal cell wall components, particularly β-glucans derived from *C. albicans*, are recognized by pattern recognition receptors, such as dectin-1, expressed on innate immune cells [34]. Activation of dectin-1 triggers downstream signaling pathways involving Syk (spleen tyrosine kinase) and CARD9 (Caspase recruitment domain-containing protein 9), leading to the production of pro-inflammatory cytokines and the differentiation of Th17 cells [35]. Furthermore, the Th17 immune axis plays a pivotal role in antifungal defense by promoting neutrophil recruitment and mucosal barrier protection [36]. However, persistent activation of this pathway, as observed in chronic fungal overgrowth, has been proposed as a potential mechanism underlying sustained mucosal inflammation and tissue injury [37].

Clinical studies have reported associations between fungal dysbiosis and inflammatory bowel disease (IBD), although the directionality and biological significance of these associations remain incompletely understood [38]. In patients with Crohn’s disease, increased abundance of *C. tropicalis* has been reported and correlated with disease severity and inflammatory activity [39]. However, these observations should be interpreted cautiously because reverse causality and confounding factors cannot be excluded. Intestinal inflammation itself, together with antibiotic exposure, dietary modifications, and immunomodulatory therapies, may independently alter fungal community composition. Similarly, *Malassezia restricta*, a lipid-dependent yeast traditionally associated with the skin microbiome, has been detected in the intestinal tract and linked to inflammatory processes [40]. Mechanistically, experimental studies suggest that *Malassezia* species may activate complement pathways and potentially amplify inflammatory signaling cascades under specific host conditions [41,42]. These findings highlight the expanding spectrum of fungal organisms implicated in gut pathology and underscore the importance of species-specific immune interactions.

Disruption of the intestinal epithelial barrier represents a critical downstream consequence of fungal overgrowth. The epithelial barrier serves as a physical and immunological interface that prevents translocation of luminal microbes and their products into the systemic circulation [43]. *C. albicans* contributes to barrier dysfunction through the production of candidalysin, a pore-forming peptide toxin that directly damages epithelial cell membranes [3]. Candidalysin induces calcium influx, mitochondrial dysfunction, and stress response pathway activation, ultimately leading to epithelial cell death and loss of barrier integrity [44].

In parallel, fungal metabolism generates acetaldehyde, a highly reactive and cytotoxic compound that further compromises epithelial integrity [45]. Acetaldehyde interferes with tight junction proteins, including occludin and claudins, and disrupts cytoskeletal organization, resulting in increased intestinal permeability [46]. In addition, acetaldehyde impairs mitochondrial respiration and promotes oxidative stress, thereby amplifying epithelial injury [47]. These mechanisms collectively contribute to the development of a “leaky gut” phenotype, which facilitates translocation of microbial products and perpetuates systemic immune activation.

In contrast to *C. albicans*, *C. glabrata* exhibits a distinct pathogenic strategy characterized by persistence rather than overt tissue invasion. This organism cannot form hyphae but compensates with enhanced adhesion to epithelial surfaces and capacity for intracellular survival within host cells [48]. *C. glabrata* is also notable for its resistance to oxidative stress and reduced susceptibility to azole antifungal agents [49]. These properties enable long-term colonization and contribute to fungal overgrowth chronicity, particularly in immunocompromised hosts.

Biofilm formation represents a central feature of fungal pathogenicity and plays a crucial role in treatment resistance. Biofilms are structured microbial communities embedded within an extracellular matrix composed of polysaccharides, proteins, and extracellular DNA [50]. *C. albicans* and *C. tropicalis* are particularly adept at forming robust biofilms that confer protection against antifungal agents and host immune responses [51]. Within biofilms, fungal cells exhibit altered metabolic states and reduced susceptibility to pharmacological interventions [52].

*C. glabrata*, although a weak biofilm former, contributes to biofilm stability through adhesive interactions and cooperative behavior within polymicrobial communities [4]. These interactions enhance the resilience of biofilms and complicate eradication efforts. Importantly, polymicrobial biofilms involving *C. tropicalis* have been identified in patients with Crohn’s disease, which provides supporting evidence for a potential mechanistic link between fungal overgrowth and chronic intestinal inflammation [39]. These biofilms not only sustain local inflammation but also serve as reservoirs for persistent infection, thereby contributing to disease recurrence and treatment failure [53].

The intestinal mycobiome encompasses a broader range of fungal organisms beyond the species most extensively studied in IFO. Although *C. albicans* remains the dominant focus of current research, accumulating evidence suggests that additional fungal taxa may contribute to intestinal dysbiosis through distinct pathogenic mechanisms. *C. parapsilosis* has been associated with enhanced epithelial adherence and biofilm formation [54], *C. krusei* exhibits intrinsic resistance to several azole antifungal agents [55], and *Debaryomyces hansenii* (formerly *C. famata*) has been implicated in impaired mucosal healing and persistent intestinal inflammation [56]. *C. auris* has recently emerged as a multidrug-resistant fungal pathogen of global clinical concern [57]. Although gastrointestinal colonization by *C. auris* has been reported and may contribute to persistent carriage and healthcare-associated transmission, direct evidence linking *C. auris* to IFO, gastrointestinal symptom generation, or chronic intestinal inflammation remains limited. Overall, the clinical significance of *C. parapsilosis*, *C. krusei*, *Debaryomyces hansenii*, and *C. auris* in IFO remains less well established than that of *C. albicans*, *C. tropicalis*, and *C. glabrata*, highlighting the need for further investigation.

In addition to pathogenic fungi, commensal organisms such as *Saccharomyces cerevisiae* may also play a role in disease through immune-mediated mechanisms. Anti-*Saccharomyces cerevisiae* antibodies (ASCA) are frequently detected in patients with Crohn’s disease and are considered a marker of immune reactivity to fungal antigens [58]. Although the precise role of *S. cerevisiae* in disease pathogenesis remains unclear, its association with immune activation suggests that even non-pathogenic fungi may contribute to dysregulated host responses under conditions of dysbiosis [59]. Importantly, not all fungal organisms exert pathogenic effects. Certain fungi may provide beneficial effects under specific conditions. For example, *Saccharomyces boulardii* has demonstrated probiotic properties, including inhibition of *Candida* biofilm formation, enhancement of epithelial barrier integrity, and modulation of host immune responses [60,61,62]. These observations suggest that the biological consequences of fungal dysbiosis are species-dependent and should not be generalized across the entire intestinal mycobiome.

Collectively, these findings indicate that IFO is a multifactorial process involving interkingdom microbial interactions, immune dysregulation, epithelial barrier disruption, and biofilm-mediated persistence. Importantly, these mechanisms are highly species-dependent, as different fungal organisms contribute to disease through distinct yet interconnected pathways. This mechanistic heterogeneity underscores the need for a more nuanced understanding of the intestinal mycobiome and its role in gastrointestinal disease.

**Table 2:** Pathophysiological Mechanisms of Intestinal Fungal Overgrowth and Species-Specific Contributions.

Pathophysiological Domain	Mechanism	Representative Species	Key Virulence Features	Molecular/Cellular Effects	Reference
Interkingdom interaction	Loss of bacterial colonization resistance	Multiple fungal species	Competitive expansion after antibiotic exposure	Reduced SCFA, altered microbial signaling	[23,24]
Morphological transition	Yeast-to-hyphal switching	*Candida albicans*	Hyphal formation, phenotypic switching	Adhesion, epithelial invasion, virulence gene activation	[25,26,27]
Microbial network disruption	Polymicrobial biofilm formation	*C. tropicalis* + bacteria	Synergistic biofilm architecture	Increased structural stability, antimicrobial resistance	[30,31,32,33]
Immune activation	β-glucan–Dectin-1–CARD9 pathway	*C. albicans*	Cell wall β-glucan exposure	Th17 differentiation, cytokine production	[34,35,36,37]
Inflammatory amplification	Complement-mediated immune activation	*Malassezia restricta*, *C. tropicalis*	Mannose-binding lectin activation	Amplified inflammatory signaling cascades	[38,39,40,41,42]
Epithelial barrier disruption	Toxin-mediated injury	*C. albicans*	Candidalysin production	Tight junction disruption, epithelial cell death	[3,44]
Metabolic toxicity	Fungal metabolite production	*C. albicans*	Acetaldehyde generation	Mitochondrial dysfunction, oxidative stress	[45,46,47]
Persistence strategy	Intracellular survival and stress resistance	*C. glabrata*	Adhesion, oxidative stress resistance	Immune evasion, reduced antifungal susceptibility	[48,49]
Biofilm-mediated resistance	Structured biofilm formation	*C. albicans*, *C. tropicalis*	Extracellular matrix formation	Reduced drug penetration, altered metabolism	[50,51,52,53]
Adhesion and biofilm persistence	Surface adhesion and biofilm formation	*C. parapsilosis*	Adhesins, biofilm formation	Persistent mucosal colonization and treatment resistance	[54]
Antifungal resistance	Intrinsic azole resistance	*C. krusei*	Reduced fluconazole susceptibility	Persistence under antifungal selective pressure	[55]
Delayed mucosal healing	Impaired epithelial restitution	*Debaryomyces hansenii* (*C. famata*)	Macrophage activation and wound-healing delay	Sustained intestinal inflammation and impaired tissue repair	[56]
Immune modulation	Antigen-driven adaptive response	*Saccharomyces cerevisiae*	ASCA antigenicity	Adaptive immune activation	[58,59]

SCFA, short-chain fatty acids; ASCA, Anti-*Saccharomyces cerevisiae* Antibodies.

## 5. Clinical Manifestations and Disease

The clinical manifestations of IFO are inherently heterogeneous and often lack disease-specific features, which contributes to the ongoing challenge of clinical recognition. The clinical implications of IFO across gastrointestinal, immunological, and systemic domains are summarized in Table 3. Patients frequently present with nonspecific gastrointestinal symptoms, including bloating, abdominal distension, excessive gas, and altered bowel habits [63]. However, these symptoms alone do not adequately capture the complexity of clinical presentations associated with fungal dysbiosis or allow for differentiation from other forms of microbiome-related disorders.

A key limitation in current clinical understanding is the tendency to interpret IFO within the framework of symptom-based disorders, particularly irritable bowel syndrome (IBS). While symptom overlap is substantial, fungal overgrowth has been proposed as a potential feature of a subset of patients with persistent symptoms and limited responses to conventional therapies, although this concept requires further validation [64]. In this context, IFO may influence disease phenotype in selected patients, but its precise clinical significance remains uncertain.

One of the distinguishing clinical features associated with fungal dysbiosis is the persistence of symptoms despite standard therapeutic interventions [7]. Patients with suspected IFO often report minimal or transient responses to treatments, such as antibiotics and dietary modifications, targeting bacterial overgrowth. This pattern of therapeutic refractoriness may reflect underlying microbial complexity, particularly the presence of biofilm-associated fungal communities that are less susceptible to conventional treatments. Consequently, the identification of treatment-resistant symptom patterns may provide an important clinical clue for the presence of fungal involvement.

Beyond symptom persistence, temporal variability in symptom expression represents another clinically relevant feature. Patients with fungal overgrowth may experience fluctuations in symptom severity that correlate with dietary intake, particularly the consumption of fermentable carbohydrates [65]. This pattern is consistent with the metabolic dependence of fungal organisms on carbohydrate substrates [66] and may help differentiate fungal-related symptoms from other gastrointestinal conditions. However, the specificity of this observation remains limited, and further studies are needed to define its diagnostic utility.

An additional layer of complexity arises from the potential for extra-intestinal manifestations. While the primary clinical presentation of IFO is gastrointestinal, a subset of patients report systemic symptoms, including fatigue, cognitive impairment, and generalized malaise [67]. These manifestations are often difficult to quantify and may overlap with functional or psychosomatic disorders. Nevertheless, these observations remain largely hypothesis-generating and raise the possibility that fungal dysbiosis may influence systemic symptoms through mechanisms involving microbial translocation and immune activation [68,69]. Although causality has not been definitively established, the recognition of these broader symptom patterns is important for comprehensive clinical assessment.

The role of fungal dysbiosis in IBD further illustrates the clinical relevance of IFO beyond functional disorders. Alterations in the intestinal mycobiome have been consistently observed in patients with Crohn’s disease, in parallel with enrichment of specific fungal taxa associated with disease activity and severity [70]. Importantly, fungal involvement in IBD appears to be context-dependent and to interact with bacterial communities and host immune responses to influence disease progression [38,71]. These observations suggest that fungal dysbiosis may function as a disease-modifying factor that contributes to interindividual variability in clinical outcomes rather than serving as an independently established pathogenic driver. Consequently, future longitudinal studies examining symptom trajectories and treatment outcomes will be essential for establishing causal relationships between fungal dysbiosis and clinical manifestations, effectively distinguishing primary drivers from secondary consequences of pre-existing mucosal inflammation.

From a clinical perspective, IFO is increasingly recognized as a heterogeneous condition encompassing a spectrum of phenotypic expressions rather than a single disease entity [70,72]. Patients may present with predominantly luminal symptoms, inflammatory features, or systemic manifestations. The identification of such subgroups has important implications for both diagnosis and treatment, as different phenotypes may respond differently to therapeutic interventions.

**Table 3:** Clinical Manifestations and Disease Implications of Intestinal Fungal Overgrowth (IFO).

Clinical Manifestation	Clinical Characteristics and Implications	References
Nonspecific gastrointestinal symptoms (bloating, abdominal distension, excessive gas, and altered bowel habits)	Symptoms are nonspecific and frequently overlap with functional gastrointestinal disorders, making clinical differentiation challenging.	[63,64]
Persistent or treatment-resistant bloating, abdominal discomfort, and altered bowel habits	Symptoms may persist despite conventional therapy, suggesting that fungal overgrowth may define a clinically relevant subset of patients with refractory gastrointestinal symptoms.	[64,65]
Symptom fluctuation associated with dietary intake	Symptom severity may fluctuate following ingestion of fermentable carbohydrates, potentially reflecting fungal metabolic activity.	[65,66]
Extra-intestinal manifestations (fatigue, cognitive impairment, and generalized malaise)	Although the primary presentation is gastrointestinal, some patients report systemic symptoms that may affect overall quality of life. These manifestations have been hypothesized to result from immune activation and microbial translocation; however, supporting evidence remains limited, and causality has not been established.	[67,68,69]
Clinical heterogeneity and distinct phenotypic expressions	IFO may represent a spectrum of disease states rather than a single uniform clinical entity, and has implications for diagnosis and treatment.	[70,72]

Another important consideration is the interaction between fungal dysbiosis and host susceptibility factors. Patients with metabolic disorders, immunological alterations, or chronic medication use may exhibit distinct clinical patterns that reflect underlying host–microbial interactions. For example, individuals with diabetes mellitus may experience more persistent or severe symptoms due to the combined effects of metabolic dysregulation and fungal proliferation [73]. Similarly, patients receiving long-term proton pump inhibitor therapy may present with upper gastrointestinal symptoms related to altered microbial colonization patterns [74]. Despite these observations, the clinical characterization of IFO remains limited by the absence of validated phenotypic criteria. Most current descriptions are based on observational studies and clinical experience rather than standardized definitions [7,64], and this lack of uniformity complicates both research and clinical practice, as it hinders the identification of patient populations that may benefit from targeted interventions.

Future research should therefore focus on developing a more refined clinical framework for IFO that incorporates symptom patterns, risk factors, microbial profiles, and treatment responses. Integration of these elements may enable the identification of clinically meaningful subtypes and facilitate more personalized approaches to management.

Taken together, the clinical manifestations of IFO reflect complex interplay between microbial activity, host factors, and environmental influences. While gastrointestinal symptoms remain the most prominent feature, the broader clinical spectrum includes treatment resistance, symptom variability, and potential systemic effects [7,64]. Recognizing this complexity is essential for advancing the diagnosis and management of IFO and for integrating fungal dysbiosis into the broader framework of gastrointestinal diseases.

## 6. Diagnostic Challenges

The diagnosis of IFO remains a significant unmet need in clinical gastroenterology due to technical limitations and conceptual ambiguity regarding what constitutes clinically relevant fungal dysbiosis [75]. A fundamental challenge lies in determining whether simple fungal colonization and pathogenic overgrowth contribute to symptom generation or disease progression [76]. Unlike bacterial overgrowth, for which quantitative thresholds have been proposed [77], no universally accepted criteria exist for defining clinically significant fungal expansion [64].

One of the key limitations of current diagnostic approaches is their inability to capture spatial heterogeneity within the gastrointestinal tract. Fungal populations in the small intestine and colon and along proximal–distal gradients differ markedly [78]. Conventional stool-based analyses, while convenient, predominantly reflect colonic microbiota and may fail to detect overgrowth occurring in the small intestine, where clinical symptoms are often generated [79,80]. This spatial disconnect introduces a systematic bias in fungal detection and may contribute to underdiagnosis.

The distinction between fungal presence and activity is another important consideration. DNA-based sequencing methods, including next-generation sequencing and metagenomics, provide detailed taxonomic profiles but cannot differentiate between viable, metabolically active organisms and inactive or transient fungal DNA [81,82]. As a result, the clinical interpretation of sequencing data remains challenging. For example, the detection of *C. albicans* in stool samples does not necessarily imply pathogenic involvement unless accompanied by evidence of metabolic activity, virulence factor expression, or host response [83].

Increasing interest in functional diagnostics that assess fungal metabolic output rather than mere presence addresses these limitations. Metabolomic profiling represents a promising approach in this regard, as it enables the detection of fungal-derived metabolites such as acetaldehyde, ethanol, and short-chain organic acids [84]. These metabolites may serve as indirect indicators of active fungal fermentation and provide a more physiologically relevant measure of fungal activity. However, the specificity of such markers creates challenges, as many metabolites are shared between fungal and bacterial metabolic pathways [85].

Host-response biomarkers represent another emerging diagnostic dimension. Measurement of immune activation markers, including cytokine profiles and fungal-specific antibody responses, may provide insight into the interactions between the host and fungal organisms [86]. For instance, the presence of ASCA reflects immune reactivity to fungal antigens but lacks specificity for IFO [59]. A comparative overview of currently available diagnostic approaches for IFO is presented in Table 4. Although multiple diagnostic modalities have been proposed, including stool-based analyses, sequencing technologies, metabolomic profiling, and host-response biomarkers, none currently demonstrates sufficient sensitivity, specificity, reproducibility, and clinical validation to serve as a standalone diagnostic standard. Each approach provides unique insights into fungal composition, activity, or host–microbial interactions; however, significant limitations remain regarding anatomical specificity, assessment of fungal viability, and clinical interpretability. These limitations underscore the need for integrated diagnostic frameworks that combine microbial detection, functional assessment, and host-response evaluation. Future diagnostic strategies may benefit from combining microbial detection with host-response signatures to define clinically relevant disease states more precisely.

Interkingdom interactions constitute an additional layer of complexity. Fungal pathogenicity is often dependent on the presence of specific bacterial communities, particularly in the context of polymicrobial biofilms [87]. Current diagnostic frameworks, which typically evaluate fungi in isolation, fail to capture these interactions [88], and thus integrative approaches that simultaneously assess bacterial and fungal communities may provide a more accurate representation of underlying dysbiosis.

From a clinical perspective, the lack of standardized diagnostic tools necessitates reliance on indirect clinical indicators. Patients with persistent gastrointestinal symptoms, particularly those refractory to conventional therapies, may represent a population more likely to exhibit fungal dysbiosis [72]. However, symptom-based diagnosis is inherently nonspecific and prone to bias [89]. This highlights the need for objective, reproducible diagnostic criteria that can be applied consistently across clinical settings.

Future advances in diagnostic technology will likely involve the integration of multi-modal data, including microbial composition, metabolic activity, and host response. Machine learning approaches may further enhance diagnostic accuracy by identifying patterns within complex datasets that are not readily apparent through conventional analysis [90]. Ultimately, the development of clinically applicable diagnostic tools will require rigorous validation in well-characterized patient populations.

## 7. Therapeutic Implications

Therapeutic management of IFO remains challenging because available treatment strategies are supported by limited clinical evidence and no evidence-based guidelines currently exist. Although antifungal agents remain the most frequently employed intervention, their use is largely guided by observational studies, expert opinion, and extrapolation from related fungal disorders rather than by randomized controlled trials specifically addressing IFO [7,64]. Fluconazole is the most commonly utilized systemic antifungal because of its favorable oral bioavailability and activity against many *Candida* species [7,64]. In studies of suspected SIFO, treatment courses of approximately two to three weeks have been associated with symptomatic improvement in selected patients [7]. However, interpretation of these findings is limited by small study populations, heterogeneous diagnostic criteria, and the absence of microbiological endpoints confirming fungal eradication [7,64]. Furthermore, systemic antifungal therapy may be associated with adverse effects, including hepatotoxicity, gastrointestinal intolerance, drug–drug interactions, and the emergence of antifungal resistance [64,91].

Nystatin has also been used as a therapeutic option, particularly when fungal overgrowth is presumed to be confined to the intestinal lumen [92]. Its minimal gastrointestinal absorption reduces the likelihood of systemic toxicity and clinically significant drug interactions [92]. Nevertheless, despite its widespread empirical use, evidence supporting its efficacy in IFO remains sparse, and robust studies evaluating long-term symptom improvement, recurrence rates, and fungal clearance are lacking [64,92]. Similar limitations apply to other antifungal agents, including itraconazole, posaconazole, and oral amphotericin B formulations, which have been proposed in selected circumstances but remain supported primarily by indirect evidence and clinical experience [7,64].

Interest has increasingly shifted toward therapeutic strategies that address the broader microbial ecosystem rather than targeting fungi alone. Emerging evidence suggests that probiotics and other microbiome-modulating interventions may influence fungal populations indirectly through restoration of bacterial communities, enhancement of mucosal barrier function, and suppression of fungal virulence traits [23,93,94,95]. Experimental studies have demonstrated inhibitory effects of probiotic organisms, including *Saccharomyces boulardii*, on Candida species and their associated biofilms [95,96]. However, clinical evidence remains insufficient to support routine use in patients with IFO, and optimal strains, treatment duration, and patient selection criteria have yet to be established.

Dietary interventions have likewise been proposed as adjunctive strategies because nutrient availability is a major determinant of fungal growth and metabolic activity [97]. Restriction of simple sugars and highly fermentable carbohydrates may theoretically limit fungal proliferation; however, direct clinical evidence supporting dietary therapy specifically for IFO remains limited [65,98]. Similarly, microbiome-targeted approaches such as fecal microbiota transplantation have generated increasing interest because restoration of bacterial communities may indirectly influence fungal ecology and interkingdom interaction [99]. Nevertheless, their effects on fungal dysbiosis remain incompletely understood, and further investigation is required before clinical recommendations can be made.

Biofilm-associated resistance represents a major barrier to effective treatment. Fungal cells within biofilms exhibit altered gene expression, reduced metabolic activity, and increased resistance to antifungal agents [100]. This state of phenotypic tolerance differs from classical resistance and is not captured by standard susceptibility testing [101]. Strategies aimed at disrupting biofilm structure or preventing its formation may therefore enhance therapeutic efficacy. Emerging approaches include the use of biofilm-disrupting agents, quorum-sensing inhibitors, and combination therapies that target multiple aspects of fungal pathogenicity [102].

Taken together, the current therapeutic landscape for IFO remains largely exploratory. While available interventions may provide symptomatic benefit in selected patients, substantial uncertainty persists regarding optimal treatment selection, duration of therapy, prevention of recurrence, and long-term clinical outcomes. The absence of standardized diagnostic criteria further complicates interpretation of therapeutic studies and highlights the need for well-designed prospective trials incorporating species-level fungal characterization, objective microbiological endpoints, adverse-event monitoring, and validated clinical outcome measures [7,64].

Importantly, therapeutic decision-making should incorporate patient-specific factors, including comorbid conditions, medication use, and baseline microbiome composition. Personalized approaches that integrate clinical, microbial, and metabolic data may offer the greatest potential for optimizing treatment outcomes [101]. As our understanding of fungal dysbiosis evolves, therapeutic strategies are likely to shift toward precision-based interventions that target specific pathogenic mechanisms.

The strength of evidence supporting the various mechanistic, clinical, diagnostic, and therapeutic aspects of IFO is summarized in Table 5. While several biological mechanisms, including fungal virulence factors, epithelial barrier disruption, and antifungal immune pathways, are supported by substantial experimental evidence, much of the current clinical literature remains observational. Consequently, confidence in the available evidence varies considerably across different domains of IFO research. Mechanistic insights are generally supported by robust experimental data, whereas evidence regarding clinical manifestations, diagnostic strategies, and therapeutic interventions remains comparatively limited and, in some cases, speculative.

## 8. Conclusions and Future Directions

The growing recognition of the intestinal mycobiome has expanded current understanding of gut dysbiosis beyond its traditional bacterial framework and has highlighted the potential contribution of fungal communities to gastrointestinal and systemic disease. Emerging evidence indicates that fungal dysbiosis may influence host physiology through multiple mechanisms, including epithelial barrier disruption, immune activation, biofilm formation, and complex interkingdom interaction [76,102,106]. Nevertheless, many of the associations described in the current literature remain observational, and the extent to which fungal alterations directly contribute to disease pathogenesis has yet to be established. Interpretation of the current literature is further complicated by substantial heterogeneity among published studies. Differences in patient selection, diagnostic methodologies, fungal detection techniques, geographic background, dietary exposures, and underlying disease states likely contribute to inconsistent findings across cohorts. These limitations highlight the need for standardized study designs and larger prospective investigations to better define the clinical significance of fungal dysbiosis [8,9,10,74,75].

Progress in the field will depend on the development of standardized diagnostic frameworks capable of distinguishing commensal colonization from clinically relevant fungal overgrowth [7,64]. Advances in sequencing technologies, metabolomics, and multi-omics approaches offer promising opportunities to characterize fungal communities with greater precision and to integrate information regarding microbial composition, metabolic activity, and host response [103,104,105]. Such approaches may ultimately facilitate identification of biologically meaningful disease phenotypes and improve diagnostic accuracy.

At the same time, important therapeutic uncertainties remain. Current management strategies rely largely on empirical antifungal therapy and microbiome-directed interventions, both of which are supported by limited clinical evidence. Future studies should therefore focus not only on clarifying the pathogenic significance of fungal dysbiosis but also on determining whether targeted therapeutic interventions result in meaningful and sustained clinical benefit. Particular attention should be directed toward species-specific mechanisms, host susceptibility factors, and the ecological interactions that shape treatment response.

Taken together, IFO should currently be regarded as an evolving clinical concept rather than a fully established disease entity. Although substantial progress has been made in understanding the intestinal mycobiome, important gaps remain in diagnostic validation, therapeutic evidence, and causal inference. Future studies integrating the mycobiome, bacteriome, and virome will be essential to clarify the contribution of fungal dysbiosis to gastrointestinal disease and to support the development of evidence-based management strategies.

## Figures and Tables

**Table 1:** Risk Factors for Intestinal Fungal Overgrowth (IFO).

Category	Factors	Mechanism	Predominant Fungal Species	Clinical Implication	Reference
Medications	Antibiotics	Disruption of bacterial colonization resistance; reduced SCFA production	*Candida albicans*, *Candida tropicalis*	Rapid fungal expansion, dysbiosis	[2,11,12]
	Proton pump inhibitors	Reduced gastric acidity and altered upper GI microbiota	*Candida* spp., *Saccharomyces* spp.	Upper GI colonization, IFO risk	[14]
Metabolic	Diabetes mellitus	Hyperglycemia enhances fungal growth, adhesion, and immune dysfunction	*Candida glabrata*, *Candida albicans*	Persistent colonization, severe symptoms	[15]
	Obesity/dyslipidemia	Altered lipid metabolism supporting fungal growth	*Malassezia restricta*	Systemic inflammation, metabolic dysregulation	[16]
Immune	Immunosuppression	Impaired antifungal immunity (Th17 dysfunction)	*Candida* spp.	Opportunistic overgrowth	[17]
	Corticosteroid use	Reduced innate immune response	*Candida* spp.	Increased infection susceptibility	[18]
GI factors	Motility disorders	Stasis promotes microbial accumulation	Mixed fungal species	Overgrowth persistence	[7]
	Reduced bile acid activity	Decreased antimicrobial effect	*Candida* spp.	Fungal survival advantage	[19]
Lifestyle	High-carbohydrate diet	Increased fermentable substrates	*Candida albicans*	Gas production, symptom exacerbation	[20]
	Alcohol consumption	Acetaldehyde accumulation and barrier damage	*Candida* spp.	Mucosal injury	[21]
Other	Chronic illness/hospitalization	Microbiome disruption and immune alteration	Mixed species	Complex dysbiosis	[22]

SCFA, short-chain fatty acids; GI, gastrointestinal.

**Table 4:** Comparative Evaluation of Current Diagnostic Approaches for Intestinal Fungal Overgrowth (IFO).

Diagnostic Method	Principle	Sensitivity	Specificity	Reproducibility	Clinical Utility	Advantages	Limitations	References
Stool-based fungal analysis (culture or sequencing)	Detection of fungal organisms in fecal samples	Not validated for IFO	Not validated for IFO	Moderate	Limited clinical utility for suspected SIFO	Non-invasive, widely available	Reflects predominantly colonic rather than small-intestinal mycobiota; cannot reliably distinguish colonization from pathogenic overgrowth	[79,80]
DNA-based sequencing (ITS sequencing, NGS, metagenomics)	Identification of fungal taxa through fungal DNA profiling	Clinical sensitivity not established	Clinical specificity not established	Moderate; affected by extraction methods, sequencing platform, and databases	Valuable research tool for mycobiome characterization	High taxonomic resolution; detects non-culturable fungi	Cannot distinguish viable from non-viable organisms; lacks validated clinical thresholds for IFO	[81,82]
Metabolomic profiling	Detection of fungal-derived metabolites (acetaldehyde, ethanol, organic acids)	Not established	Not established	Limited	Investigational	Provides information regarding fungal metabolic activity rather than simple presence	Many metabolites overlap with bacterial metabolism; no validated diagnostic cutoffs currently exist	[45,84,85]
Host-response biomarkers (ASCA, cytokines, antifungal antibodies)	Assessment of host immune responses to fungal antigens	For ASCA in Crohn’s disease: approximately 40–70%	For ASCA in Crohn’s disease: approximately 80–95%	Moderate	Potential adjunctive marker of host–fungal interaction	Reflects biologically relevant immune activation	ASCA performance data derive from Crohn’s disease rather than IFO; no validated biomarker exists specifically for IFO diagnosis	[58,59]
Small intestinal aspirate fungal culture *	Quantitative fungal culture from duodenal/jejunal aspirates	Not established	Not established	Limited due to sampling variability	Currently the most direct method for detecting viable fungi in suspected SIFO	Species identification and viability assessment possible	Invasive, expensive, no universally accepted diagnostic threshold; sensitivity and specificity have not been validated	[7,64]

* In the study by Erdogan and Rao, fungal overgrowth was identified in 38 of 150 patients with unexplained gastrointestinal symptoms (25.3%) using small bowel aspirate cultures; however, no sensitivity, specificity, or validated diagnostic threshold was established. SIFO, small intestinal fungal overgrowth; ITS, Internal Transcribed Spacer; NGS, Next-Generation Sequencing; ASCA, Anti-Saccharomyces cerevisiae Antibodies.

**Table 5:** Strength of Evidence Supporting Major Concepts in Intestinal Fungal Overgrowth (IFO).

Topic	Main Finding	Predominant Evidence Type	Evidence Strength *	References
Candida virulence mechanisms	Hyphal transformation and candidalysin promote epithelial invasion and inflammation	In vitro, animal studies	Strong	[3,25,26,27,28,44]
Fungal immune signaling pathways	Dectin-1–CARD9–Th17 signaling regulates antifungal immunity and inflammatory responses	In vitro, animal, human immunology studies	Strong	[34,35,36,37]
Polymicrobial biofilms and fungal–bacterial interactions	Biofilm formation and interkingdom interaction enhance persistence, antimicrobial tolerance, and inflammatory potential	In vitro, animal, limited human studies	Moderate	[30,31,32,33]
Epithelial barrier dysfunction	Fungal virulence factors and metabolites may impair barrier integrity and increase intestinal permeability	Experimental and observational studies	Moderate	[3,44,45,46,47]
Fungal dysbiosis in inflammatory bowel disease	Altered fungal communities are associated with intestinal inflammation and disease activity	Animal and observational human studies	Moderate	[41,42,43,44,70,71]
IBS-like gastrointestinal symptoms	Fungal overgrowth has been associated with bloating, abdominal discomfort, and altered bowel habits	Observational studies and case series	Low–Moderate	[7,63,64,72]
Extra-intestinal manifestations	Fatigue, cognitive dysfunction, and malaise have been proposed as possible manifestations of fungal dysbiosis	Case reports and hypothesis-generating studies	Speculative	[67,68,69]
Diagnostic biomarkers and multi-omics approaches	Sequencing, metabolomics, and host-response biomarkers show diagnostic potential	Observational and proof-of-concept studies	Low	[81,82,83,84,103,104,105]
Antifungal therapy	Antifungal agents may improve symptoms in selected patients, although evidence remains limited	Small clinical studies and observational studies	Low	[7,91,92]
Probiotics, dietary interventions, and microbiome-targeted therapies	Microbiome-directed strategies may modulate fungal communities, but clinical evidence remains limited	Experimental and limited clinical studies	Low	[23,60,61,62,65,66,93,94,95,96,97,98,99]

* Evidence strength was qualitatively assessed based on study design, consistency of findings, degree of mechanistic validation, and availability of human clinical data. Strong evidence indicates consistent findings across mechanistic, animal, and/or human studies. Moderate evidence indicates supportive but incomplete evidence with limited clinical validation or unresolved causal relationships. Low evidence indicates predominantly observational, preliminary, or small-scale clinical data. Speculative evidence indicates hypothesis-generating findings supported by limited empirical evidence. IBS, irritable bowel syndrome.

## Data Availability

No new data were created or analyzed in this study. Data sharing is not applicable to this article.
